# Incidental Discovery and Removal of an Appendiceal Mucocele in a Patient With Acute Cholecystitis

**DOI:** 10.7759/cureus.102962

**Published:** 2026-02-04

**Authors:** Harrison Hunter, Annabel Barber, Daman Samrao

**Affiliations:** 1 General Surgery, Kirk Kerkorian School of Medicine, University of Nevada, Las Vegas, Las Vegas, USA; 2 Pathology, Kirk Kerkorian School of Medicine, University of Nevada, Las Vegas, Las Vegas, USA

**Keywords:** acute cholecystitis, appendiceal mucocele, cavernous hemangioma, incidental appendiceal lesion, laparoscopic appendectomy, pseudomyxoma peritonei, robotic cholecystectomy, serum tumor markers

## Abstract

Appendiceal mucoceles (AMs) are a rare clinical entity among appendiceal tumors. They are characterized by dilation of the appendix and accumulation of mucus within the lumen. These lesions are often discovered incidentally during imaging or surgery for unrelated abdominal complaints. We report a case of a 55-year-old male who presented with right upper quadrant pain due to acute cholecystitis. An AM and a hepatic lesion were incidentally identified on abdominal CT. The patient underwent a robotic-assisted laparoscopic cholecystectomy and appendectomy, with pathology confirming a benign AM and acute cholecystitis. A coincidental hepatic lesion was also biopsied and diagnosed as a hemangioma. Although rare and usually benign, AMs have the potential to rupture and cause the potentially fatal condition pseudomyxoma peritonei. They may also become malignant, act as lead points for intussusception, or cause bowel obstruction due to mass effect. This case highlights the importance of a comprehensive abdominal evaluation and demonstrates the value of prompt surgical excision of AMs to prevent complications.

## Introduction

An appendiceal mucocele (AM) is a clinical term referring to abnormal distension of the appendix due to accumulation of mucinous material within its lumen [1-3]. AMs represent an uncommon subset of appendiceal pathologies, accounting for only 0.3-0.7% of cases [1]. This condition has significant clinical implications because of its potential for malignant transformation or peritoneal dissemination if ruptured. Between 11% and 20% of AMs are malignant mucinous adenocarcinomas [4,5], and 16% of AMs present with pseudomyxoma peritonei. Up to 50% of patients with malignant mucinous adenocarcinoma of the appendix will present with pseudomyxoma peritonei [5].

The term “mucocele” encompasses simple retention cysts, mucosal hyperplasia, mucinous cystadenoma, and mucinous cystadenocarcinoma [1]. AMs are most frequently identified in middle-aged to older adults, with a reported peak incidence in the fifth to seventh decades of life. There is a slight female predominance, and mucinous neoplastic subtypes are the most common [1,4]. AMs arise from luminal obstruction of the appendix, leading to mucus accumulation. This accumulation may result from inflammation or from benign or malignant processes [1,4].

Clinically, AMs are often asymptomatic and are frequently discovered incidentally during imaging for unrelated abdominal complaints. On physical examination, a palpable, mobile right lower quadrant mass may be noted. These lesions can become painful and mimic appendicitis if rupture or infection occurs [1-5]. Imaging with ultrasound, CT, and MRI is key to diagnosing AM. Characteristic findings include a well-defined, cystic, low-attenuation mass with mural calcifications and a luminal diameter typically between 1.2 and 2.0 cm. Fat stranding may indicate rupture and associated inflammation [6].

The following case describes the incidental discovery and surgical management of an AM in a patient presenting with right upper quadrant pain secondary to acute cholecystitis. This report highlights the frequently incidental nature of AM discovery and discusses the rationale for prompt surgical removal and accurate pathological identification.

## Case presentation

A 55-year-old male with a past medical history of type 2 diabetes mellitus and hypertension presented to the ED with right upper quadrant and epigastric pain that began 12 hours earlier. He reported that the pain initially started in his upper back but later localized to the epigastric region and right upper quadrant. He described the pain as sharp and constant and denied associated nausea, vomiting, diarrhea, or constipation. He reported chills but denied fever, jaundice, or recent weight loss. There was no history of similar episodes.

The patient denied tobacco, alcohol, or illicit drug use. Family history was noncontributory. His surgical history included only the excision of a benign left neck mass 32 years prior. Current medications included metformin, glipizide, and lisinopril. He had no known drug allergies. A timeline of hospital care is summarized in Table 1.

**Table 1:** Timeline of care during hospitalization

Time point	Clinical event
Hospital day 0	Admission to the ED
Hospital day 1	Surgical procedure performed
Hospital days 2-4	Postoperative monitoring by the medicine and surgery teams
Hospital day 4	Ultrasound-guided liver biopsy
Hospital day 4	Discharged home in stable condition
Postoperative week 4	Outpatient postoperative follow-up

Physical examination

The patient was alert and in mild distress from pain. Vital signs were notable for elevated blood pressure (176/81 mmHg), a heart rate of 80 bpm, and an afebrile temperature of 98.2 °F. Abdominal examination revealed tenderness in the epigastric and right upper quadrant regions without rebound, guarding, or palpable mass.

Laboratory evaluation

The patient presented with leukocytosis, thrombocytosis, hyperglycemia, and mild anemia. Liver enzymes were within normal limits on admission but became slightly elevated over the course of the day. Laboratory values throughout the patient’s hospitalization are presented in Table 2, Table 3, and Table 4. Table 2 shows values on admission, Table 3 shows values immediately before surgery, and Table 4 shows values at discharge. Of note, the elevated liver enzymes in Table 4 likely reflect hepatic manipulation during the operation.

**Table 2:** Laboratory evaluation on admission ALK, alkaline phosphatase; ALT, alanine aminotransferase; AST, aspartate aminotransferase

Test	Result	Reference range
White blood cell count	12.48 × 10³/µL	4.0-11.0 × 10³/µL
Hemoglobin	11.1 g/dL	Male: 13.5-17.5 g/dL; female: 12.0-16.0 g/dL
Platelet count	424 × 10³/µL	150-400 × 10³/µL
CO₂ (bicarbonate)	18 mmol/L	22-29 mmol/L
Glucose	174 mg/dL	70-99 mg/dL
AST	17 U/L	10-40 U/L
ALT	17 U/L	7-56 U/L
ALK	81 U/L	44-147 U/L

**Table 3:** Laboratory evaluation preoperatively ALK, alkaline phosphatase; ALT, alanine aminotransferase; AST, aspartate aminotransferase

Test	Result	Reference range
White blood cell count	13.27 × 10³/µL	4.0-11.0 × 10³/µL
Hemoglobin	10.0 g/dL	Male: 13.5-17.5 g/dL; female: 12.0-16.0 g/dL
Platelet count	365 × 10³/µL	150-400 × 10³/µL
CO₂ (bicarbonate)	22 mmol/L	22-29 mmol/L
Glucose	171 mg/dL	70-99 mg/dL
AST	38 U/L	10-40 U/L
ALT	50 U/L	7-56 U/L
ALK	78 U/L	44-147 U/L

**Table 4:** Laboratory evaluation on discharge ALK, alkaline phosphatase; ALT, alanine aminotransferase; AST, aspartate aminotransferase

Test	Result	Reference range
White blood cell count	9.8 × 10³/µL	4.0-11.0 × 10³/µL
Hemoglobin	9.6 g/dL	Male: 13.5-17.5 g/dL; female: 12.0-16.0 g/dL
Platelet count	365 × 10³/µL	150-400 × 10³/µL
CO₂ (bicarbonate)	23 mmol/L	22-29 mmol/L
Glucose	153 mg/dL	70-99 mg/dL
AST	72 U/L	10-40 U/L
ALT	111 U/L	7-56 U/L
ALK	129 U/L	44-147 U/L

To evaluate for potential malignancy of the mucocele, serum carbohydrate antigen 19-9 (CA 19-9) levels were measured postoperatively. The serum tumor marker CA 19-9 was 11.1 U/mL (<35.0 U/mL), which is within the normal range.

Imaging 

**Figure 1:**
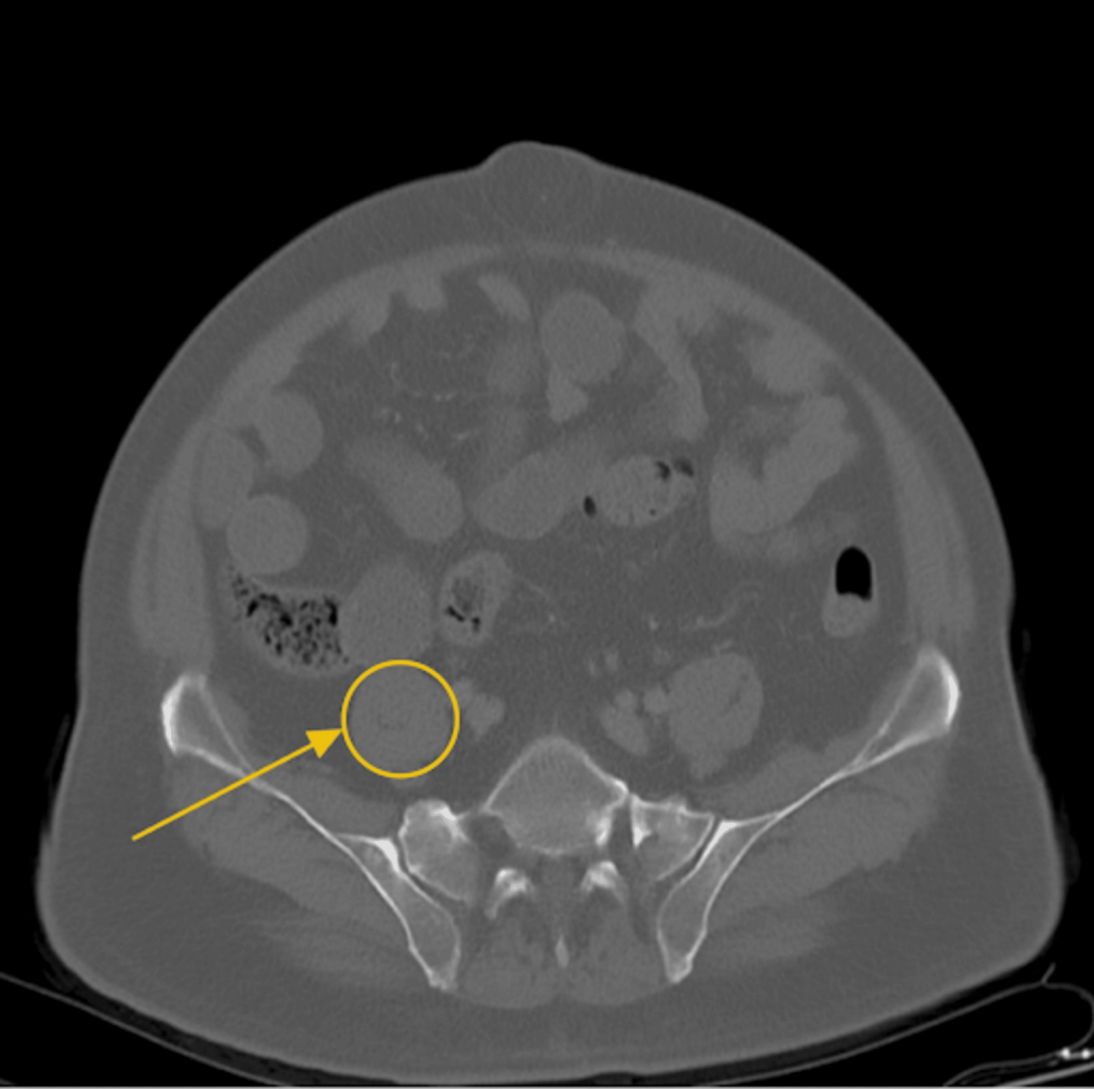
AM seen attached to the cecum on CT with contrast (transverse view) Dense tissue at the end of the cecum (yellow arrow and circle) indicates the incidental mass. AM, appendiceal mucocele

**Figure 2:**
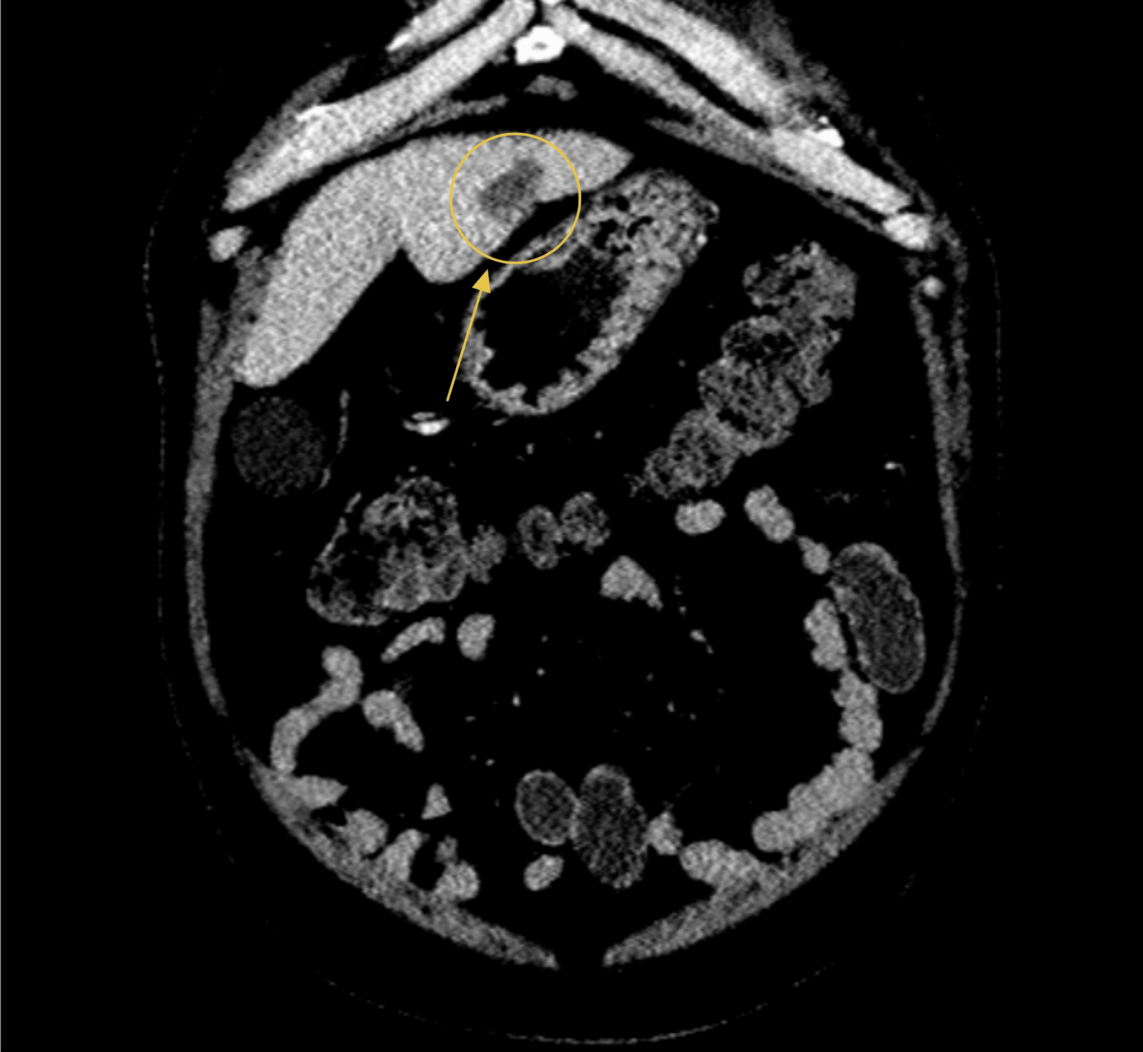
Hypodense hepatic lesion in the anterior portion of the left hepatic lobe on CT

**Figure 3:**
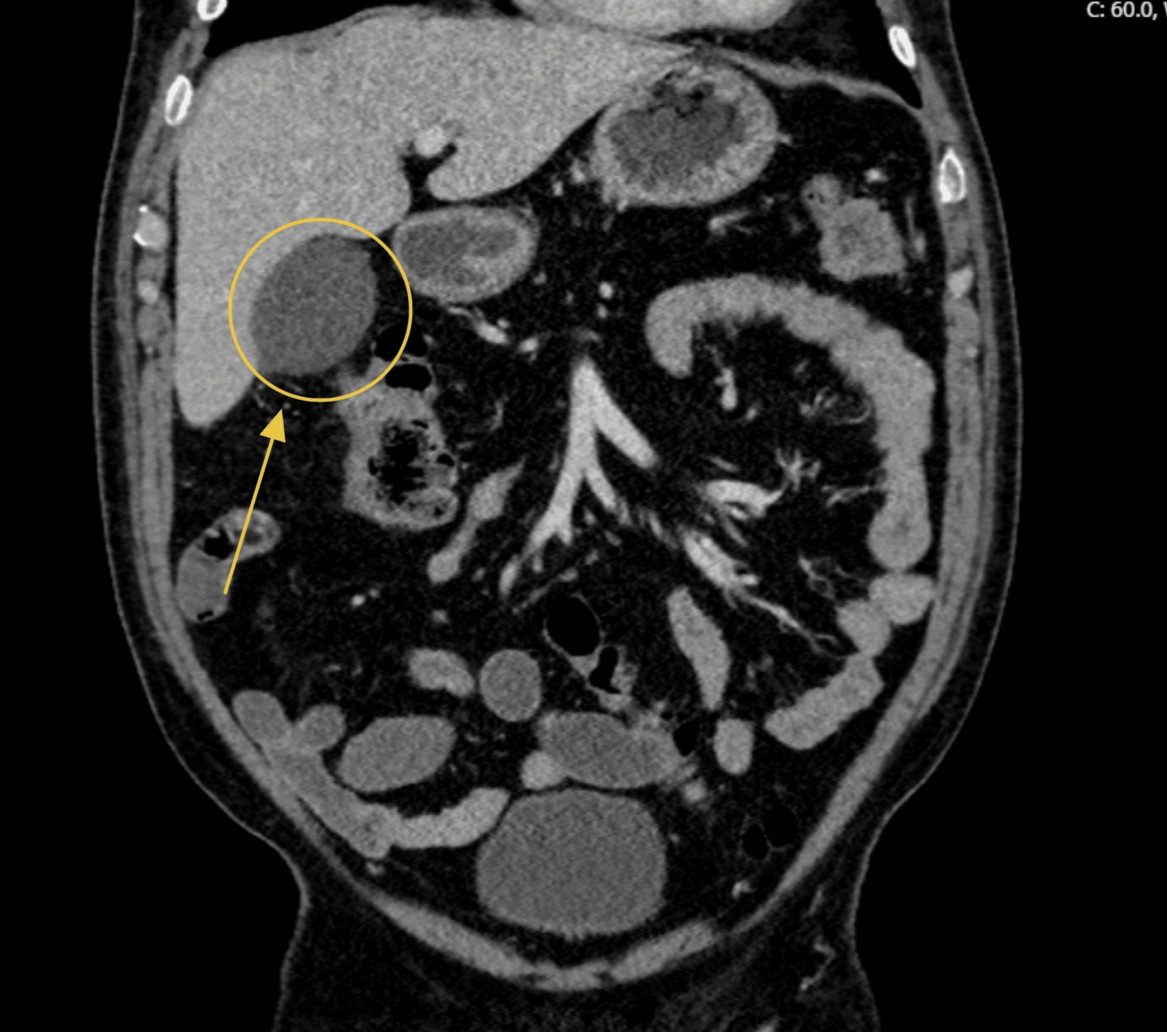
Distended gallbladder on CT

An abdominal CT scan was obtained in the ED. The first evidence of the incidental mucocele was identified as a fluid-filled, blind-ending tubular structure arising from the cecum with mural calcifications, consistent with an AM (Figure 1). Scattered diverticula were noted throughout the colon. Additionally, a 2.4-cm hypodense hepatic lesion (HU 39) was seen in the anterior portion of the left hepatic lobe, raising concern for possible metastatic disease (Figure 2). The CT also demonstrated a distended gallbladder without evidence of pericholecystic fluid (Figure 3).

**Figure 4:**
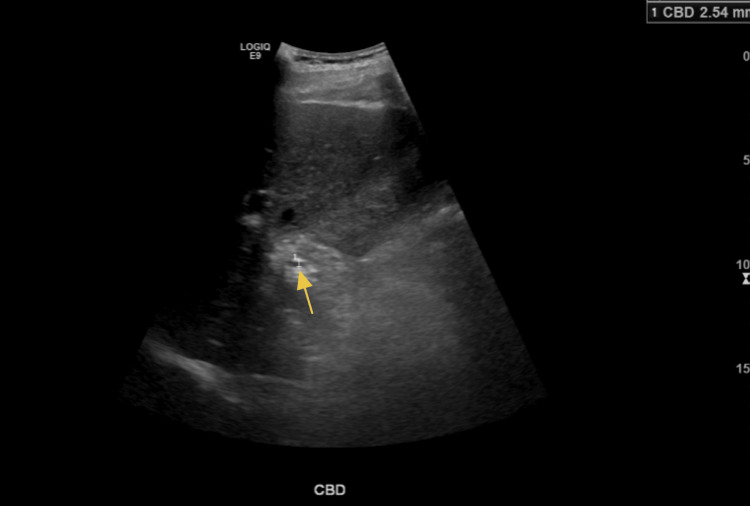
Common bile duct measuring normal size at 2.5 mm

**Figure 5:**
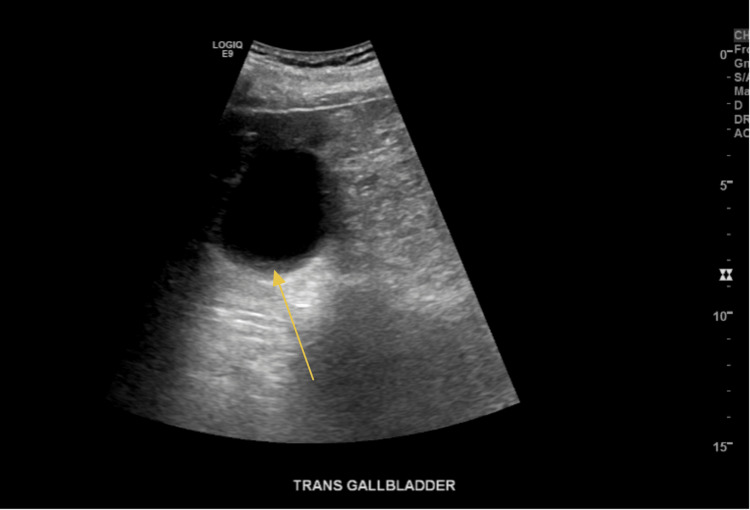
Sludge in the gallbladder (transverse view)

**Figure 6:**
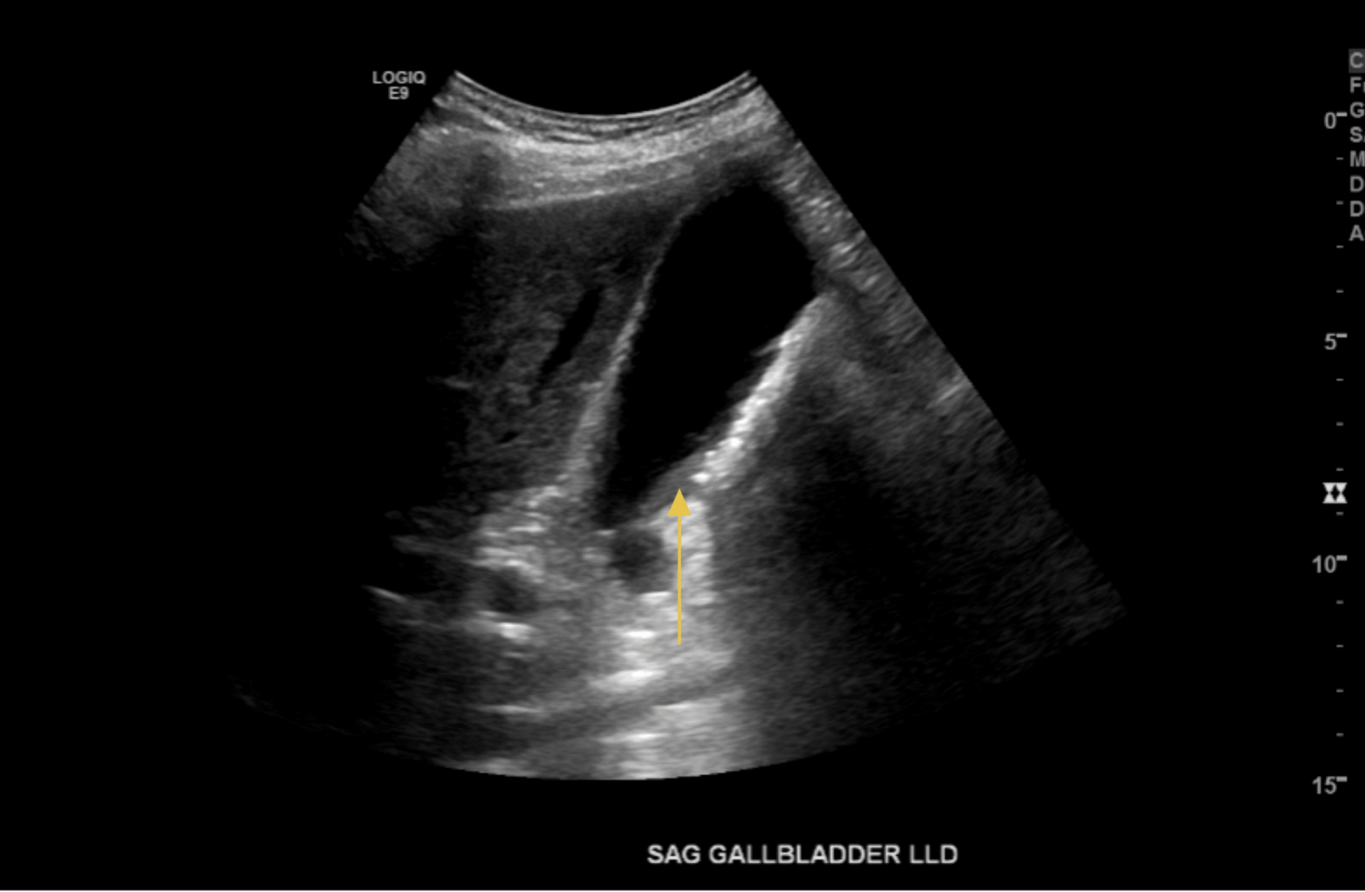
Sludge in the gallbladder (sagittal view)

A right upper quadrant ultrasound was subsequently performed to further evaluate the distended gallbladder seen on CT. The common bile duct measured 2.5 mm in diameter, which is within normal limits (Figure 4). Ultrasound demonstrated the presence of sludge without visible gallstones and a normal gallbladder wall thickness of 1 mm (Figure 5, Figure 6). No pericholecystic fluid was identified. The ultrasound did not detect the 2.4-cm hepatic lesion visualized on CT (Figure 2).

**Figure 7:**
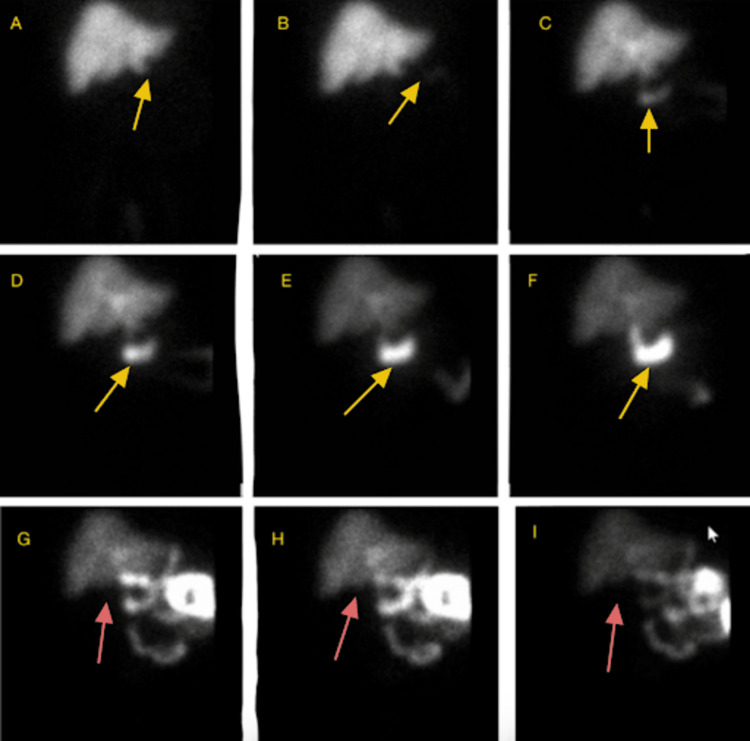
HIDA scan Panels A-F show the sequential flow of tracer through the liver and bile ducts (yellow arrows). Tracer first enters the common bile duct (best seen in panels B and C) and continues to the duodenum (panels C-F). Panels G-I were obtained after morphine administration, which constricts the sphincter of Oddi and causes tracer accumulation in the bile ducts and gallbladder. No tracer is seen entering the gallbladder (red arrows, panels G-I), indicating cystic duct obstruction. HIDA, hepatobiliary iminodiacetic acid

A hepatobiliary iminodiacetic acid scan (Figure 7) confirmed acute cholecystitis, as the gallbladder was not visualized throughout the entire study, both before and after intravenous morphine administration, consistent with cystic duct obstruction.

**Figure 8:**
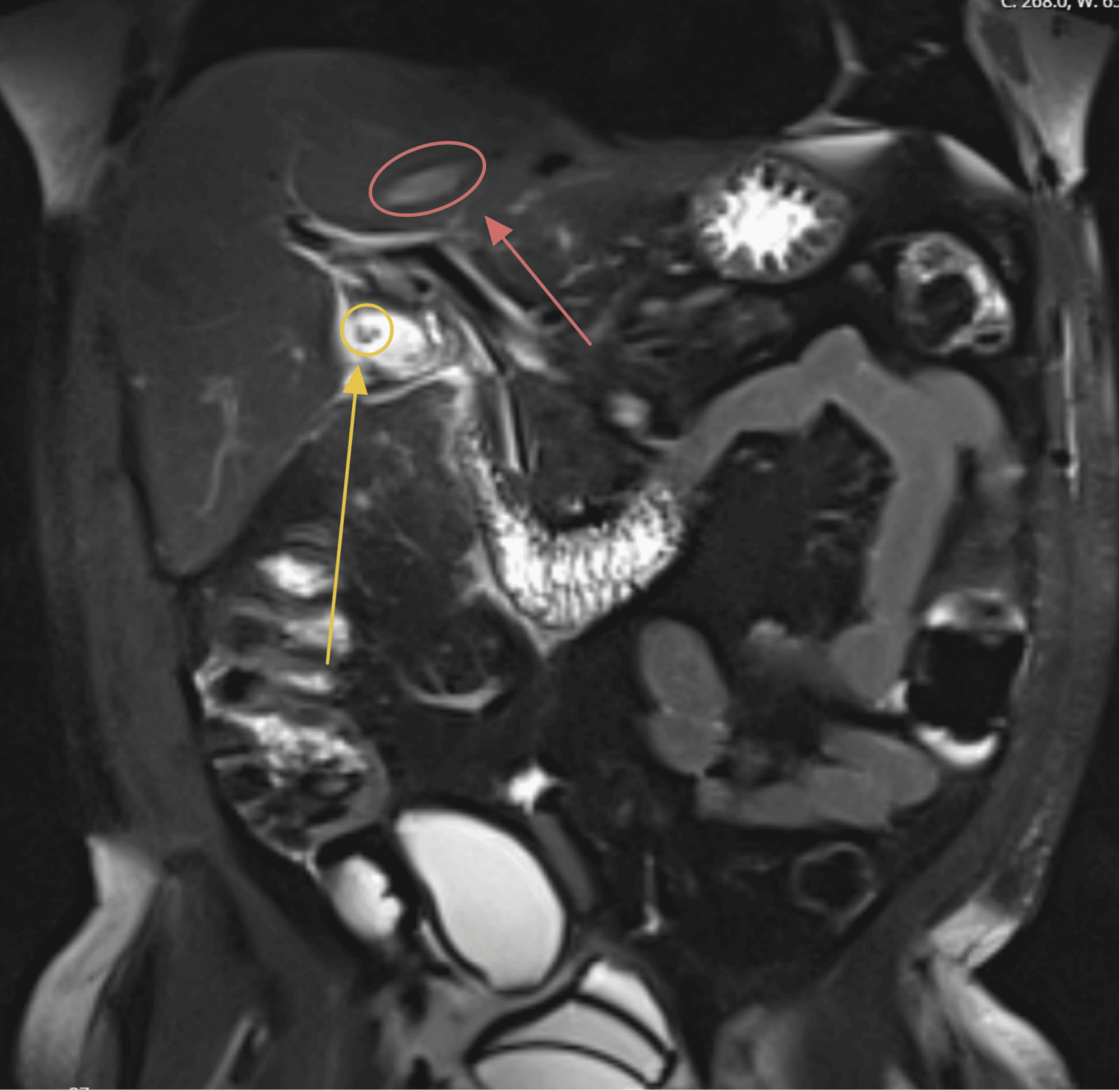
MR abdomen with arrows and a yellow circle indicating gallstones near the outlet of the gallbladder The hepatic lesion in the left lobe of the liver is circled and indicated in red.

**Figure 9:**
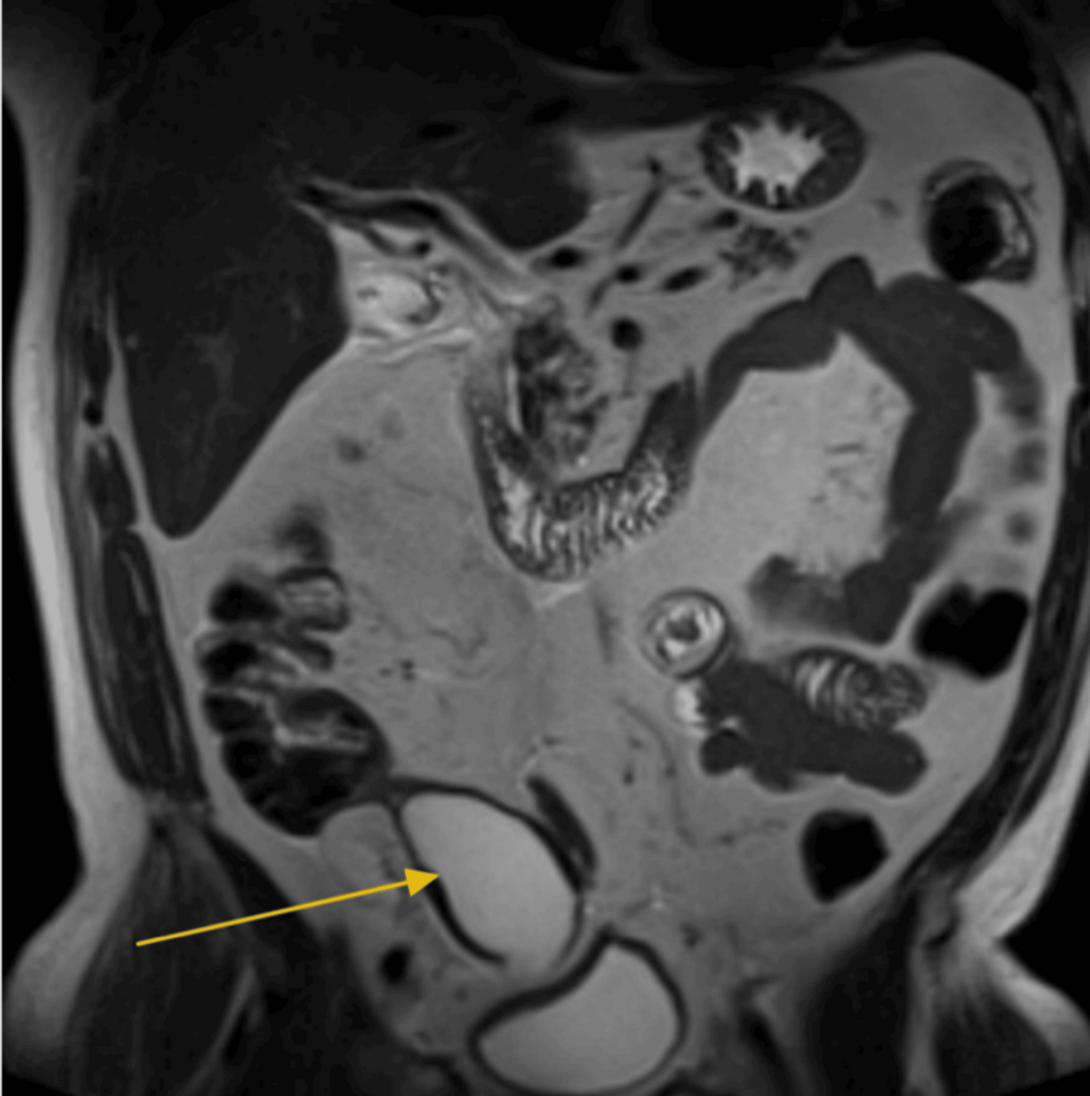
MR abdomen with contrast (coronal view) showing the AM attached to the cecum Dense tissue at the end of the cecum (yellow arrow) indicates the appendiceal mass. AM, appendiceal mucocele

The final imaging ordered in the workup of this patient was an MRI of the abdomen with and without contrast (Figure 8, Figure 9). The findings also demonstrated an approximately 2.4-cm hepatic lesion (Figure 8, red arrow). Radiology recommended obtaining a biopsy, as the lesion posed potential neoplastic risk. Imaging further reinforced the differential diagnosis of acute cholecystitis, as multiple stones were identified in the gallbladder neck region (Figure 8, yellow circle). The appendiceal mass noted on CT (Figure 1) was again visualized on MRI (Figure 9).

Clinical presentation, laboratory findings, and imaging were consistent with acute cholecystitis. Based on these findings, a robotic-assisted laparoscopic cholecystectomy with concurrent appendectomy was planned.

Management and operative course

**Figure 10:**
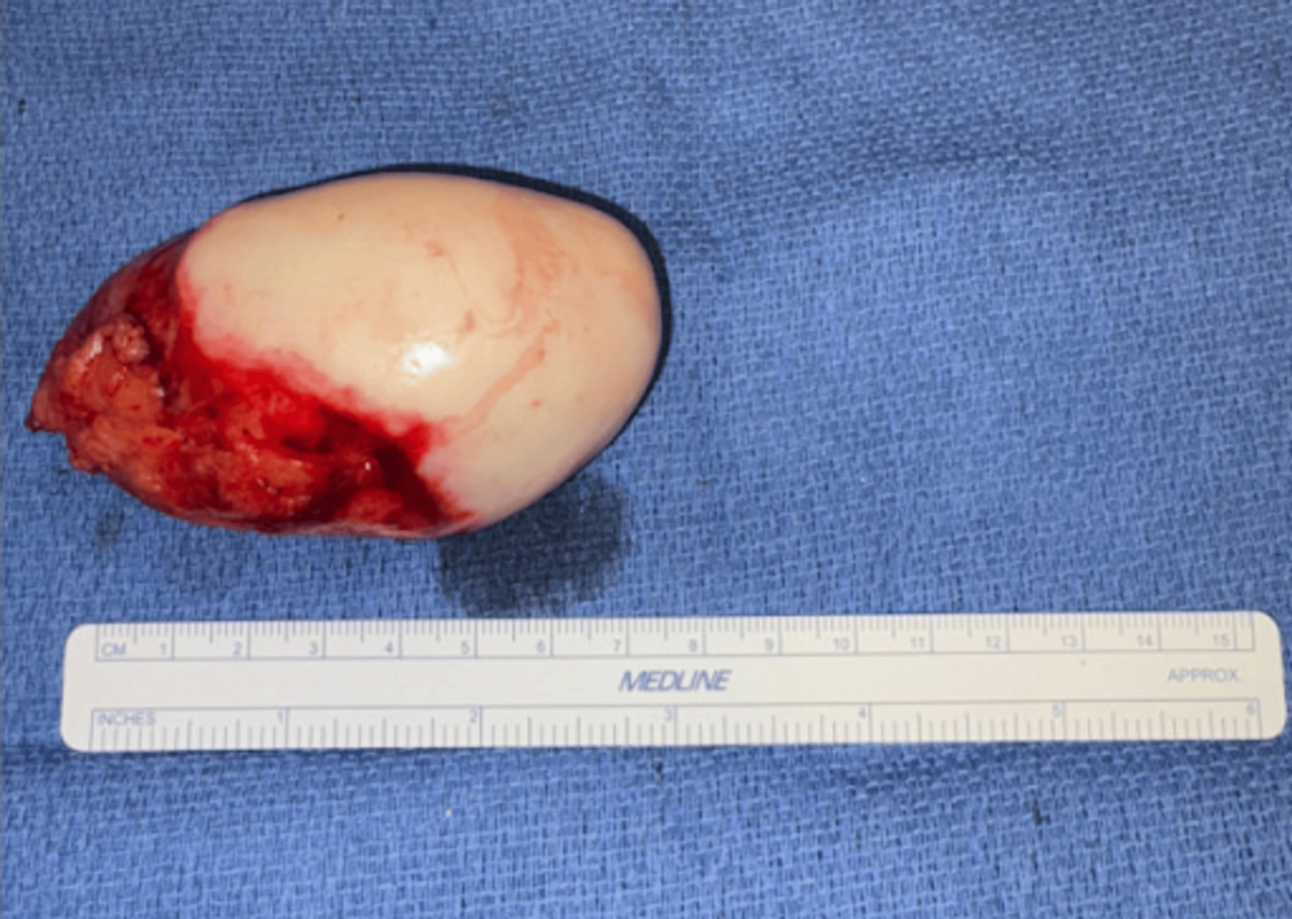
Excised mucocele Dilated appendix measuring 8.0 cm in length × 2.0 cm in height × 5.0 cm in width, consistent with an AM. The smooth, attenuated epithelium is intact and shows no evidence of rupture. AM, appendiceal mucocele

The patient provided consent and underwent a robotic-assisted laparoscopic cholecystectomy and appendectomy without intraoperative complications. Intraoperative findings included a distended, inflamed gallbladder with focal gangrenous areas and an enlarged, firm, tan-white appendix measuring 8.0 cm in length × 2.0 cm in height × 5.0 cm in width, consistent with a mucocele (Figure 10). The lumen of the mucocele was secured with a linear stapler to prevent spillage of contents into the abdomen.

**Figure 11:**
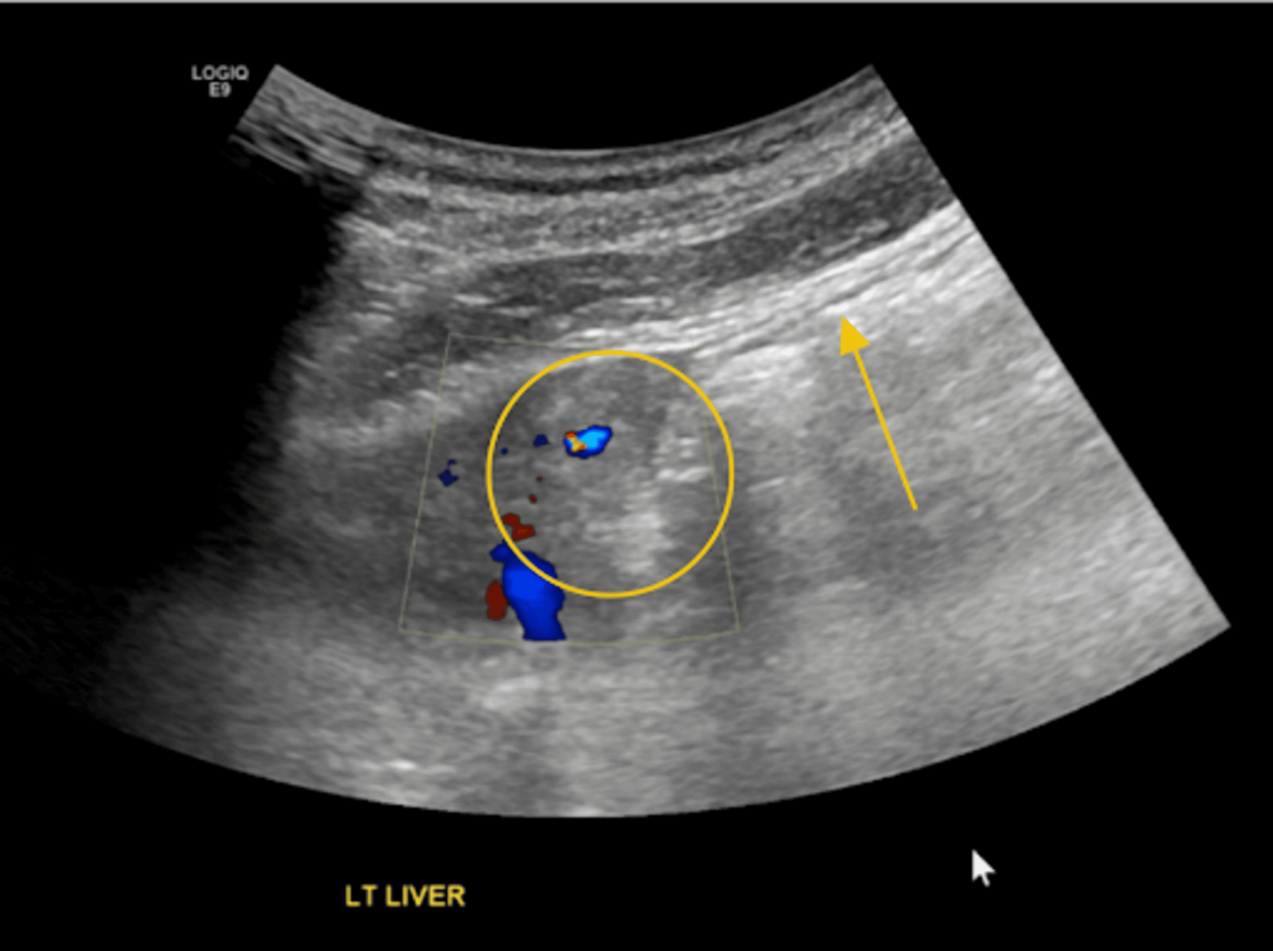
Ultrasound-guided liver biopsy A 19-gauge coaxial needle was advanced to the margin of the left hepatic mass. The needle (yellow arrow) is shown within the hepatic mass (yellow circle).

On postoperative day 3, after the patient’s leukocytosis had resolved (Table 3), he underwent an ultrasound-guided percutaneous liver biopsy (Figure 11) to evaluate the indeterminate hepatic lesion (Figure 2 and Figure 8, red arrow and circle). The postoperative course was unremarkable, and the patient was discharged home on postoperative day 5 in stable condition. At a three-week outpatient follow-up, the patient reported improvement in incisional pain. Examination revealed well-healed incisions without signs of infection. No additional labs or imaging were performed at that visit.

Pathology findings 

Histologic examination of the gallbladder revealed acute and chronic cholecystitis with focal gangrenous changes and cholelithiasis. The appendix was notably dilated and contained abundant intraluminal cloudy tan mucinous material with attenuated epithelium, consistent with an AM. No evidence of dysplasia, malignancy, or perforation was identified, supporting a benign lesion. A core biopsy of the liver demonstrated a benign vascular lesion consistent with a cavernous hemangioma, with immunohistochemistry showing CD31 positivity and cytokeratin AE1/AE3 negativity.

## Discussion

AMs are categorized into three groups: simple (retention) mucoceles, mucosal hyperplasia, and neoplastic mucoceles. Simple mucoceles are defined by luminal obstruction and mucus accumulation without epithelial atypia or proliferation. Mucosal hyperplasias involve increased mucus production with epithelial proliferation but remain benign. Neoplastic mucoceles include three subcategories: low-grade and high-grade appendiceal mucinous neoplasms (LAMN and HAMN) and adenocarcinoma. LAMN and HAMN exhibit varying degrees of atypia but do not invade surrounding tissues. Increasing atypia is characterized by prominent nuclei and increased mitotic figures, and the distinction between LAMN and HAMN is based on the extent of these features. Mucinous adenocarcinomas are malignant, invade surrounding tissues, and have metastatic potential. They pose a high risk of rupture, which can lead to pseudomyxoma peritonei [7,8].

Tumor markers such as CA 19-9 and cancer antigen 125 (CA 125) are measured in the patients’ serum to assist in detecting malignant transformation [9]. Elevated levels may indicate mucinous neoplasms or pseudomyxoma peritonei. In our case, the CA 19-9 was within normal limits, supporting a benign diagnosis.

While tumor markers are helpful in diagnosis, histopathological evaluation of the surgical specimen remains the gold standard for definitive classification. Benign structures, for example, LAMN, typically show columnar cells with stratification and abundant mucin, with normally appearing papillary folds surrounding the mucosa. Malignant structures demonstrate architectural distortion of normal papillary structures with infiltration into the muscularis [10].

The incidental discovery of an AM is clinically important due to the risks of rupture and malignant transformation. Early recognition and surgical resection are essential. In this patient, CT findings described a “fluid-filled blind-ending tubular structure off the cecum” with “internal calcifications,” which is characteristic of an AM [6]. The absence of fat stranding or wall irregularity supported a benign lesion.

Treatment depends on the lesion subtype and the presence of perforation. For unperforated mucoceles without surrounding invasion, surgical removal, as performed in this case, is the standard of care. Intraoperative handling must be meticulous to prevent rupture and mucin spillage, which can lead to pseudomyxoma peritonei. If rupture or malignancy is identified, oncologic evaluation and follow-up imaging are indicated.

This case highlights several key learning points. AMs are rare but clinically significant due to their potential for rupture and malignancy. Clinicians should maintain awareness of incidental discoveries of insidious pathologies, even in the presence of more symptomatic conditions such as acute cholecystitis. Additionally, CT findings of a cystic, low-attenuation, blind-ending tubular structure with mural calcification attached to the cecum should raise suspicion for an AM. Prompt and sterile removal without spillage is essential for curative treatment. Following excision, specimens should undergo histological evaluation, and patients should undergo serum testing for CA 19-9 and CA 125 to assess malignancy risk.

## Conclusions

AMs, although rare, represent an important diagnostic and surgical entity, ranging from benign retention cysts to invasive mucinous adenocarcinomas. Malignant variants carry a substantial risk of rupture and peritoneal dissemination; therefore, thorough imaging review, careful surgical excision, appropriate histological analysis, and tumor marker testing are crucial for proper management and prevention of complications. Early recognition and removal offer the best clinical outcomes.
